# CagA orchestrates eEF1A1 and PKCδ to induce interleukin-6 expression in *Helicobacter pylori*-infected gastric epithelial cells

**DOI:** 10.1186/s13099-020-00368-3

**Published:** 2020-07-03

**Authors:** Shaohan Xu, Xiaoqian Wu, Xiaoyan Zhang, Chu Chen, Hao Chen, Feifei She

**Affiliations:** 1 Key Laboratory of Gastrointestinal Cancer, Ministry of Education, Fujian Medical University, 1 Xue Fu North Road, Fuzhou, Fujian 350122 People’s Republic of China; 2 Key Laboratory of Tumor Microbiology, Department of Medical Microbiology, Fujian Medical University, Fuzhou, Fujian 350122 People’s Republic of China; 3 First Affiliated Hospital of Fujian Medical University, Fuzhou, Fujian 350001 People’s Republic of China

**Keywords:** CagA, eEF1A1, Interleukin-6, PKCδ, p-STAT3^S727^, Gastric adenocarcinoma

## Abstract

**Background:**

*Helicobacter pylori* colonises the stomach of approximately 50% of the global population. Cytotoxin-associated gene A protein (CagA) is one of the important virulent factors responsible for the increased inflammation and increases the risk of developing peptic ulcers and gastric carcinoma. The cytokine interleukin-6 (IL-6) has particularly important roles in the malignant transformation of gastric and intestinal epithelial cells as it is upregulated in *H. pylori*-infected gastric mucosa. In this study, we investigated the underlying mechanisms of CagA-induced IL-6 up-regulation during *H. pylori* infection. AGS cells, a human gastric adenocarcinoma cell line, lacking eEF1A1 were infected with CagA^+^*H. pylor*i (NCTC11637), CagA^−^*H. pylor*i (NCTC11637Δ*cagA*), or transduced by Ad-*cagA*/Ad-GFP. The expression and production of IL-6 were measured by quantitative real-time reverse transcription polymerase chain reaction and enzyme-linked immunosorbent assay, respectively. The interactions among CagA, eukaryotic translation elongation factor 1-alpha 1 (eEF1A1), protein kinase Cδ (PKCδ), and signal transducer and activator of transcription 3 (STAT3) were determined by western blot or co-immunoprecipitation.

**Results:**

During *H. pylori* infection, CagA-M (residues 256‒871aa) was found to interact with eEF1A1-I (residues 1‒240aa). NCTC11637 increased the expression of IL-6 in AGS cells compared with NCTC11637Δ*cagA* whereas knockdown of eEF1A1 in AGS cells completely abrogated these effects. Moreover, the CagA-eEF1A1 complex promoted the expression of IL-6 in AGS cells. CagA and eEF1A1 cooperated to mediate the expression of IL-6 by affecting the activity of p-STAT^S727^ in the nucleus. Further, CagA-eEF1A1 affected the activity of STAT3 by recruiting PKCδ. However, blocking PKCδ inhibited the phosphorylation of STAT3^S727^ and induction of IL-6 by CagA.

**Conclusions:**

CagA promotes the expression of IL-6 in AGS cells by recruiting PKCδ through eEF1A1 in the cytoplasm to increase the phosphorylation of STAT3^S727^ in the nucleus. These findings provide new insights into the function of CagA-eEF1A1 interaction in gastric adenocarcinoma.

## Background

Gastric adenocarcinoma is the second leading cause of cancer-related death worldwide and is believed to result from *Helicobacter pylori* infection and subsequent inflammation [1, 2]. However, infection with the CagA^+^*H. pylori* strain reportedly increases the risk of gastric cancer compared to infection with CagA^−^*H. pylori* [3, 4]. It is generally thought that, as a bacterial oncoprotein, CagA plays a key role in *H. pylori*-induced gastric cancer [5, 6] as it affects the expression and function of key proteins involved in oncogenic or tumour suppressor signalling pathways via several molecular mechanisms such as direct binding or interaction, phosphorylation of vital signalling proteins, and methylation of tumour suppressor proteins [7, 8]. Moreover, CagA may disrupt the balance of the gp130-activated SHP2/ERK and JAK/STAT pathways thus promoting peptic ulceration and gastric cancer in gp130^757FF^ mice [9].

Recent studies have demonstrated that the proinflammatory cytokine interleukin (IL)-6 plays an important role in the tumour progression and metastasis of multiple cancers [10–12], including gastric cancer [13, 14]. In *H. pylori*-infected gastritis patients, IL-6 levels are significantly increased [15–17]. Song et al. reported that both *H. pylori* and its toxin stimulate IL-6 expression in gastric epithelial cells [18]. This signalling pathway is mediated through protein kinase C (PKC), protein tyrosine kinase, and nuclear factor kappa-beta (NF-κB) activation, and also involves an intracellular calcium-and dexamethasone-sensitive mechanism [19]. However, the underlying mechanism of CagA-induced IL-6 expression is still poorly understood.

eEF1A1 is one of two isoforms of the alpha subunit of the elongation factor-1 complex and has many non-classical functions such as regulating cell cycle, proliferation and apoptosis [20–22]. A recent study suggests that eEF1A1 is vital for the expression of IL-6 mediated by human oncostatin-M (OSM) [23]; however, currently there are very little information on the role of eEF1A1 in gastric cancer. Interestingly, our previous yeast two-hybrid studies found that CagA may interact with YWHAE (tyrosine 3-monooxygenase/tryptophan 5-monooxygenase activation protein, epsilon polypeptide) and eEF1A1 [24]. Therefore, we hypothesized that eEF1A1 might contribute to the IL-6-inducing mechanism of CagA. Herein, we aimed to investigate whether the CagA-eEF1A1 interaction could promote the expression of IL-6 in AGS cells and to characterize the underlying mechanism for new insights into the development of novel strategies targeting pathological IL-6 expression driven by *H. pylori*.

## Results

### CagA interacts with eEF1A1

**Fig. 1:**
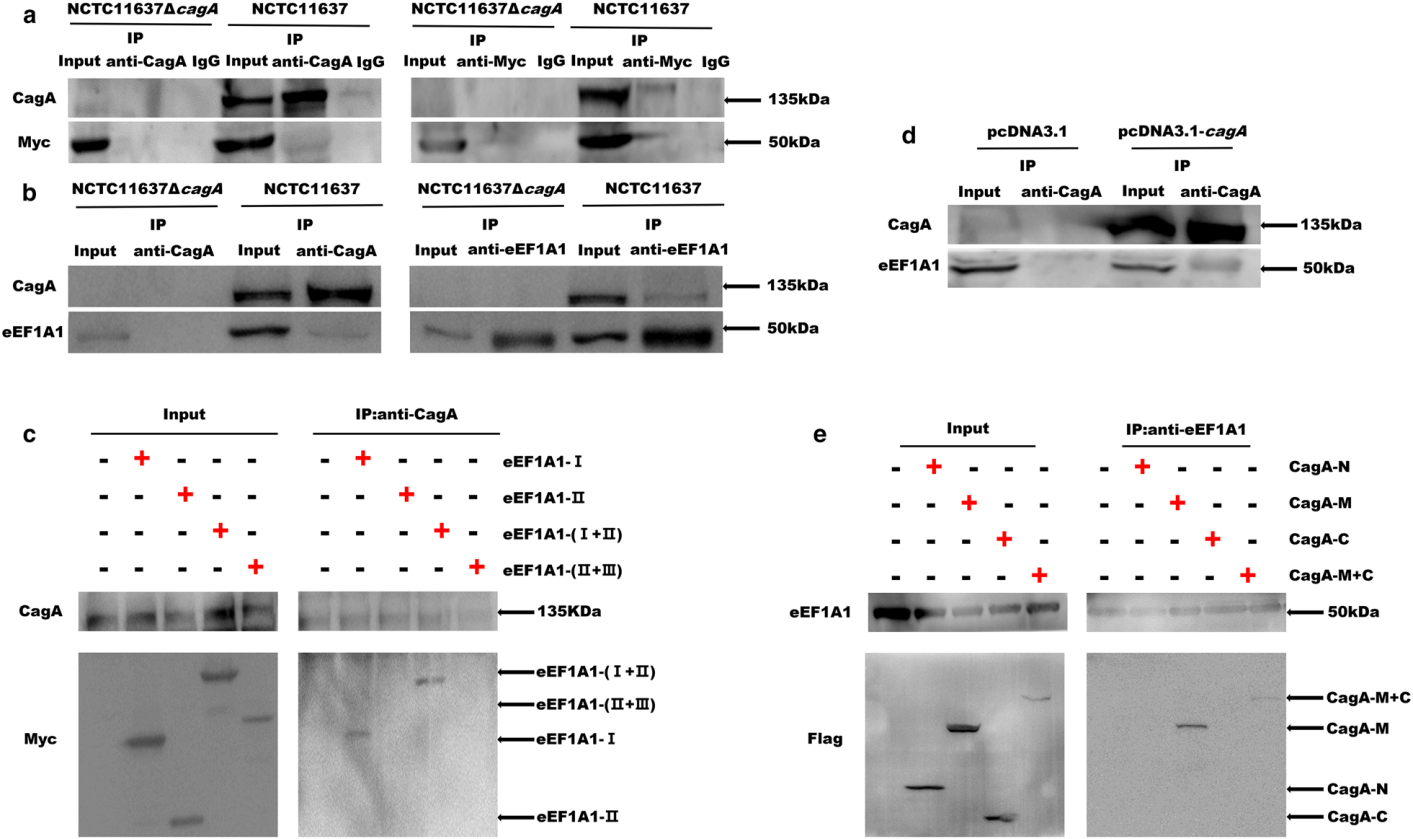
CagA interacts with eEF1A1. **a** CagA co-immunoprecipitated with exogenously expressed eEF1A1 in AGS cells. Precipitates were analyzed by western blotting (WB) using anti-CagA and anti-Myc antibodies. **b** CagA co-immunoprecipitated with endogenous eEF1A1 in AGS cells. Precipitates were analyzed by WB with anti-CagA and anti-eEF1A1 antibodies. **c** CagA co-immunoprecipitated with exogenous eEF1A1-I in AGS cells. Precipitates were analyzed by WB with anti-CagA and anti-Myc antibodies. **d** CagA co-immunoprecipitated with endogenous eEF1A1 in Cos-7 cells. Precipitates were analysed by WB with anti-CagA and anti-eEF1A1 antibodies. **e** CagA-M co-immunoprecipitated with endogenous eEF1A1 in Cos-7 cells. Precipitates were analysed by WB with anti-Flag and anti- eEF1A antibodies

As an important *H. pylori* virulence agent, CagA can form complexes with many cellular proteins and dysregulate signalling pathways via the type IV secretion system, which causes inflammation and even tumours [25–27]. In the present study, we investigated whether CagA interacted with endogenous eEF1A1. To this end, eEF1A1-overexpressing AGS cells or the control cells were infected with NCTC11637**/**NCTC11637Δ*cagA*. The co-immunoprecipitation (Co-IP) results showed that CagA had a physical contact with both exogenous and endogenous eEF1A1 (Fig. 1a, b). Moreover, structural models of eEF1A1 and CagA have revealed that each distinct surface cluster of sequence variation affords unique potential functions to the eEF1A1 or CagA proteins [28, 29]. Therefore, to comprehensively investigate whether CagA interacts with eEF1A1 after infection and to determine the interaction between CagA and eEF1A1 in a spatiotemporal manner, we constructed truncated plasmids tagged with Myc such as pcDNA3.1-*eEF1A1*-I, pcDNA3.1-*eEF1A1*-II, pcDNA3.1-*eEF1A1*-I + II, and pcDNA3.1-*eEF1A1*-II + III. AGS cells with overexpression of each segment of eEF1A1 were infected with NCTC11637. The Co-IP results showed that CagA interacted with exogenous eEF1A1-I (Fig. 1c).

To further verify that CagA could interact with eEF1A1, we optimised the CagA gene sequence to improve the expression of the prokaryotic protein CagA in eukaryotic cells. We constructed a series of Flag-tagged truncated *cagA* expressing vectors such as pcDNA3.1-*cagA*-N, pcDNA3.1-*cagA*-M, pcDNA3.1-*cagA*-C, and pcDNA3.1-*cagA*-M + C. COS-7 cells were then transfected with the truncated plasmid overexpressing each segment of CagA. Co-IP results showed that CagA could physically interact with eEF1A1 (Fig. 1d). Furthermore, we found that CagA-M and CagA-M + C but not CagA-N or CagA-C co-immunoprecipitated with eEF1A1 (Fig. 1e).

### CagA-eEF1A1 complex promotes the expression of IL-6 in AGS cells

**Fig. 2:**
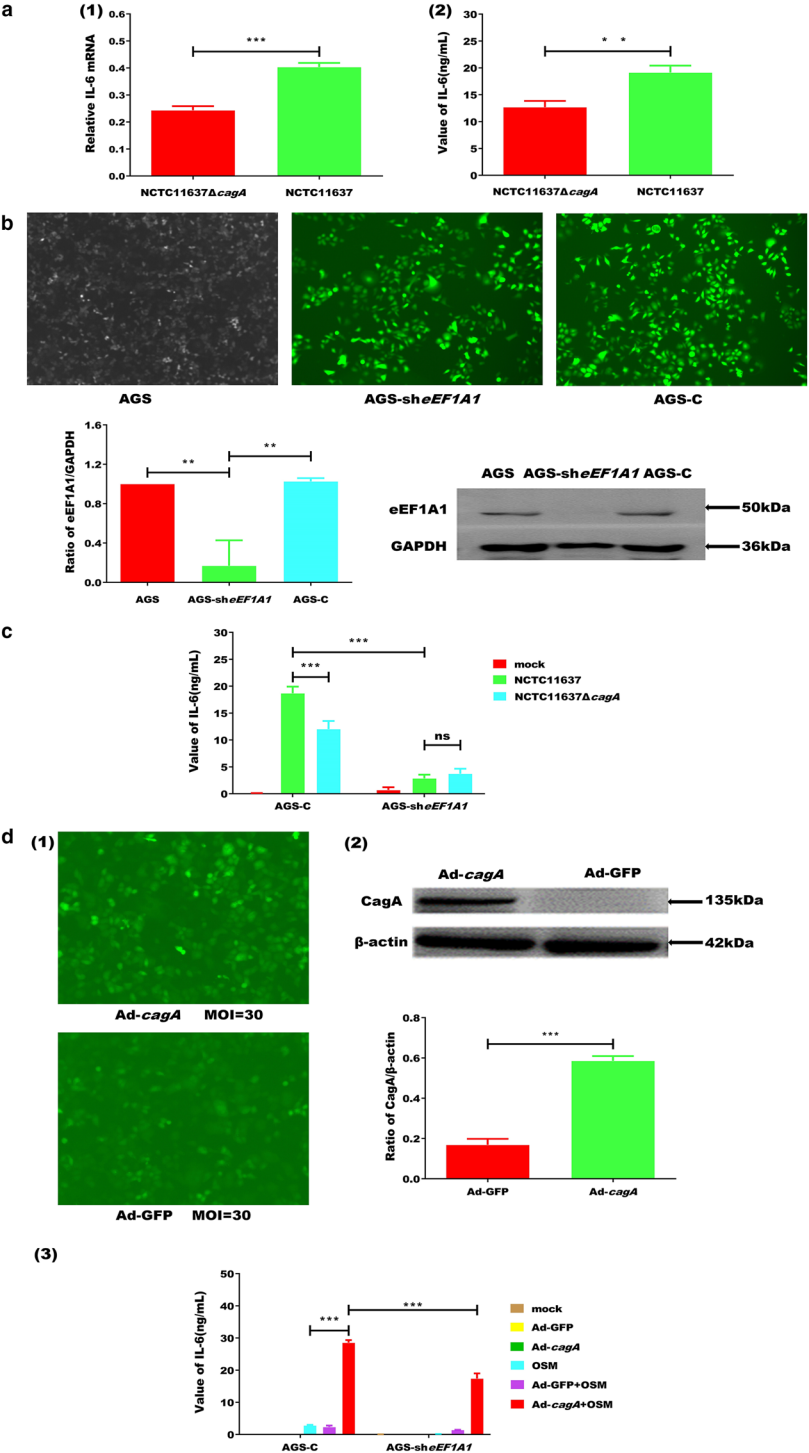
CagA and eEF1A1 co-mediate the expression of IL-6. **a** The comparison of IL-6 RNA (1) and IL-6 (2) in AGS cells that infected with NCTC11637 Δ*cagA* or NCTC11637 bacterial inoculums (MOI = 100, 16 h). **b** Fluorescence microscope image and eEF1A1 level using western blotting (WB) analysis of AGS cells that transduced with LV-eEF1A1-RNA or LV-CON077-RNA for 48 h. **c** The levels of IL-6 in AGS-C and AGS-sh*eEF1A1* cells that infected with blank or NCTC11637 or NCTC11637Δ*cagA*. **d** Fluorescence microscope image of AGS cells transduced with Ad-*cagA* (CagA fusion virus particles) or Ad-GFP (a negative control) (1), CagA was detected by WB analysis (2); The levels of IL-6 in AGS-C and AGS-sh*eEF1A1* cells that transduced with blank or Ad-GFP or Ad-*cagA* treated by OSM (100 ng/mL, 24 h) or the drug vehicle (3). Error bar represents the SDs of triplicate experiments. Statistical analysis was performed using Student’s t-tests. *P < 0.05; **P < 0.01; ***P < 0.001

Since eEF1A1 is crucial for IL-6 mRNA expression and IL-6 secretion [23], we next investigated the role of CagA in this process. We compared the level of IL-6 in AGS infected with NCTC11637 or NCTC11637Δ*cagA* and found that CagA increased the levels of IL-6 mRNA and protein in AGS cells (Fig. 2a). Next, we knocked down eEF1A1 in AGS cells (Fig. 2b) and infected AGS-C and AGS-sh*eEF1A1* (eEF1A1 knocked down) with NCTC11637/NCTC11637Δ*cagA*. Cells infected with NCTC11637Δ*cagA* had lower levels of IL-6 than did those infected with NCTC11637, and IL-6 expression was further repressed by eEF1A1 knockdown (Fig. 2c).

**Fig. 3:**
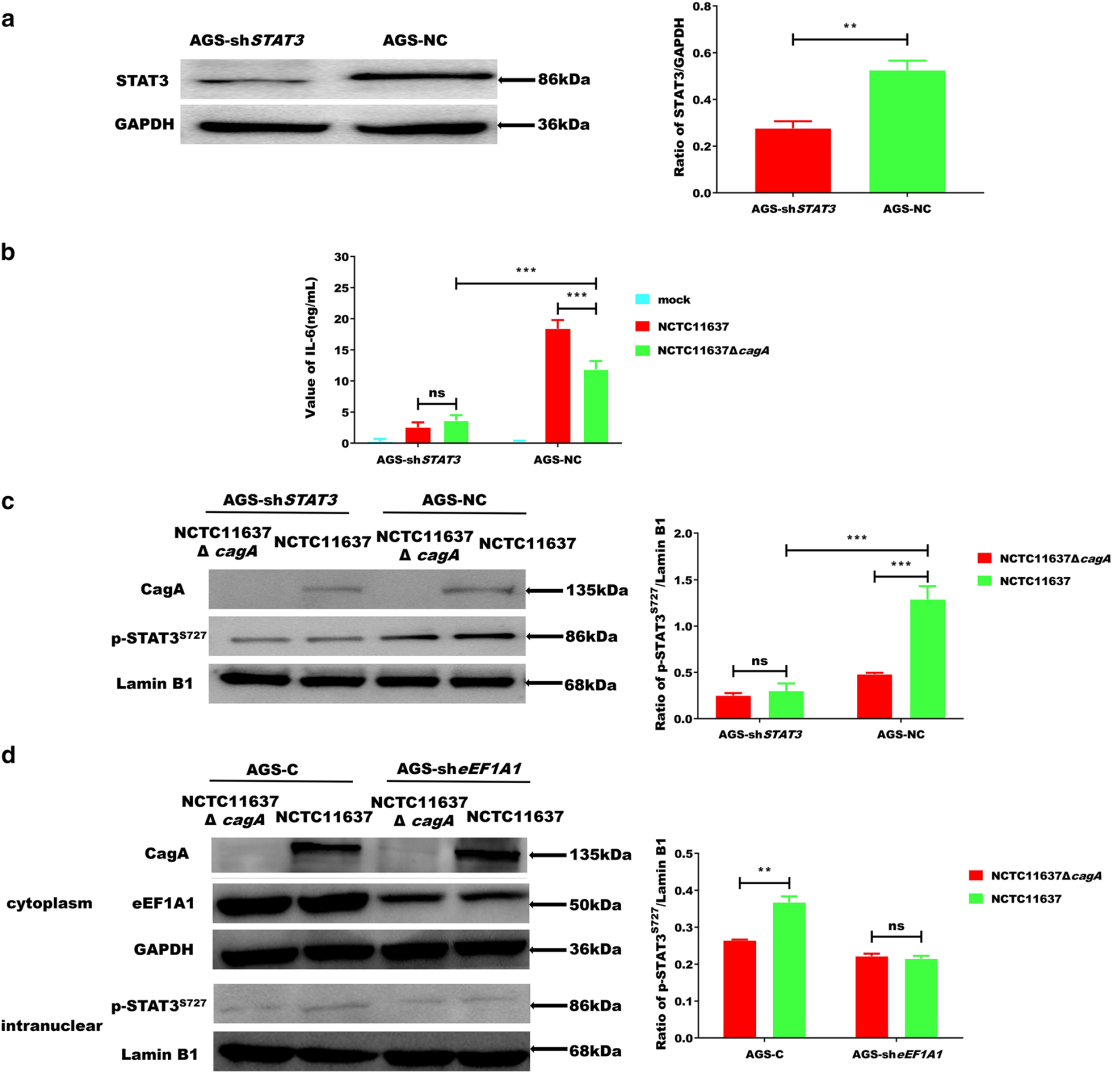
CagA-eEF1A1 complex induces IL-6 through STAT3 (S727) phosphorylation. **a** Western blot analysis to assess STAT3 in AGS-sh*STAT3* cells (STAT3 knockdown by transduced with Lv-*STAT3*-shRNA viruses) and AGS-NC cells (negative control, transduced with Lv-con-shRNA viruses). **b** The levels of IL-6 in AGS-sh*STAT3* and AGS-NC infected with blank or NCTC11637 or NCTC11637Δ*cagA*. **c** The levels of p-STAT3^S727^ in AGS-sh*STAT3* and AGS-NC cells infected with NCTC11637Δ*cagA* or NCTC11637 using WB analysis. **d** The levels of p-STAT3^S727^ in AGS-C and AGS-sh*eEF1A1* infected with NCTC11637Δ*cagA* or NCTC11637 using WB analysis. Error bar represents the SDs of triplicate experiments. Statistical analysis was performed using Student’s t-tests. *P < 0.05; **P < 0.01; ***P < 0.001

To validate these findings, we transduced AGS-C and AGS-sh*eEF1A1* cells with CagA fusion virus particles (Ad-*cagA*) or a negative control (Ad-GFP) (Fig. 2d, 1–2). After treatment with human OSM, supernatants were collected and IL-6 levels were quantified by enzyme-linked immunosorbent assay (ELISA). We observed that the level of IL-6 was higher in AGS-C cells with Ad-*cagA* than in AGS-C cells with Ad-GFP. Furthermore, the level of IL-6 was higher in AGS-C cells transduced with Ad-*cagA* than in the other AGS-sh*eEF1A1* cell lines. No differences were observed in the IL-6 levels between the AGS-sh*eEF1A1* cell lines transduced with Ad-*cagA* and AGS-sh*eEF1A1* cell lines transduced with Ad-GFP (Fig. 2d, 3).

### CagA and eEF1A1 cooperate to mediate the expression of IL-6 by affecting the activity of p-STAT^S727^ in the nucleus

Previous studies have shown that the production of IL-6 in gastric epithelial cells is affected by the phosphorylation of STAT3 in the nucleus, and that p-STAT3^S727^ can interact with NF-κB-p65 to affect the production of IL-6 and development of mesangial cancers [30, 31]. Knockdown of STAT3 in AGS cells (Fig. 3a) revealed that CagA no longer promoted IL-6 expression (Fig. 3b). Simultaneously, we observed that CagA regulated the phosphorylation of STAT3 (Fig. 3c), which suggested that *H. pylori* infection may promote the production of IL-6 by increasing the phosphorylation of STAT3 (S727). We also found that CagA failed to increase p-STAT3^S727^ in the nucleus following eEF1A1 deletion (Fig. 3d). These results further suggest that CagA and eEF1A1 cooperate to mediate the expression of IL-6 by affecting the phosphorylation of STAT3 (S727) in the nucleus.

### CagA-eEF1A1 affects the activity of STAT3 by recruiting PKCδ

**Fig. 4:**
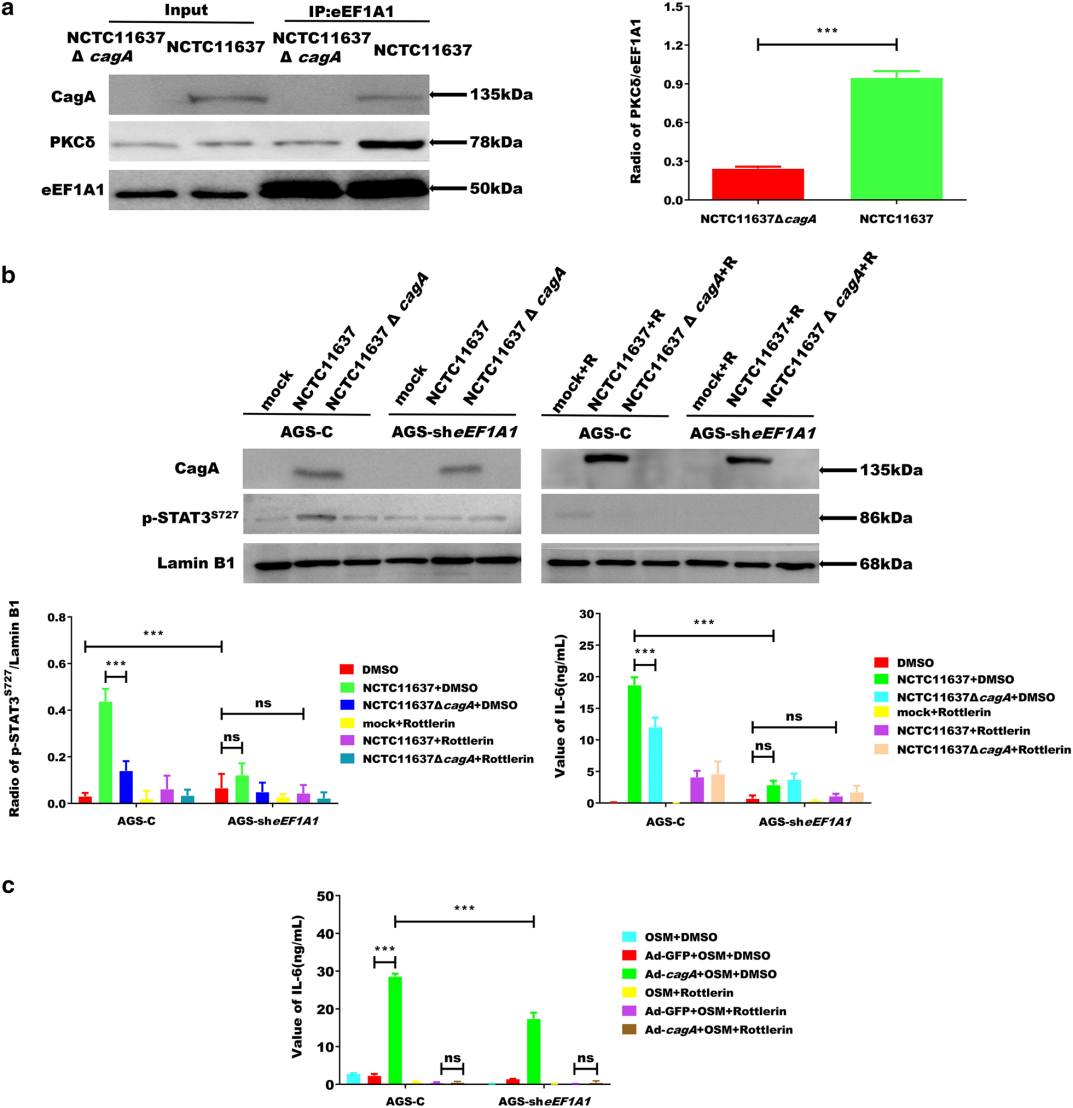
CagA-eEF1A1-PKCδ complex increases the amount of p-STAT3^S727^ in the nucleus. **a** Co-immunoprecipitation analysis of the precipitates where more PKCδ was recruited by eEF1A1 with the presence of CagA. **b** Rottlerin (6 µM for 30 min) treated AGS-C and AGS-sh*eEF1A1* cells were infected with NCTC11637 or NCTC11637Δ*cagA* bacterial inoculums (MOI = 100) for detection of p-STAT3^S727^ and CagA by western blot analysis, and the level of IL-6 in the culture supernatant. **c** The level of IL-6 of the supernatant of rottlerin (6 µM for 30 min) treated AGS-C and AGS-sh*eEF1A1* cells transduced with Ad-*cagA* or Ad-GFP (MOI = 50). Error bar represents the SDs of triplicate experiments. *P < 0.05; **P < 0.01; ***P < 0.001

PKCδ, an important member of the PKC family, can regulate IL-6 production [18]. Additionally, both eEF1A1 and STAT3 are substrates of PKCδ, and PKCδ regulates gp130 activity via non-classical SH2-mediated STAT3 binding and phosphorylation at serine 727 [32]. PKCδ can regulate STAT3 phosphorylation to affect its DNA binding and transcriptional activities [33]. Furthermore, PKCδ phosphorylates eEF1A1 at threonine 431 [33], potentially affecting the non-canonical role of eEF1A1 in IL-6 receptor signalling. Therefore, to determine whether PKCδ was involved in the CagA pathway, we performed Co-IP and found that more PKCδ was recruited by eEF1A1 when CagA was present (Fig. 4a).

To further confirm that the PKCδ-CagA-eEF1A1 complex regulated IL-6 production, AGS-sh*eEF1A1* and AGS-C cells were treated with the PKCδ inhibitor rottlerin (5,7-dihydroxy-2,2-dimethyl-6-(2,4,6-trihydroxy-3-methyl-5-acetylbenzyl)-8-cinnamoyl-1,2-chromine) prior to *H. pylori* infection. Results showed that the ability of CagA-eEF1A1 to regulate IL-6 production was significantly inhibited, and STAT3 phosphorylation was also reduced. Additionally, the expression of IL-6 was blocked by rottlerin (Fig. 4b). Measurement of IL-6 levels in AGS-sh*eEF1A1* and AGS-C cells treated with rottlerin prior to transduction with Ad-*cagA* and AD-GFP was taken to validate the blocking effects of rottlerin. The ability of CagA-eEF1A1 to regulate IL-6 production was significantly inhibited (Fig. 4c).

## Discussion

In the *H. pylori*-infected gastric mucosa, the important pathophysiological event is the induction of an inflammatory response, which is stimulated by inflammatory cytokines produced by epithelial cells [34]. IL-6 and its receptor have been shown to be important factors in *H. pylori* gastric related cancer. The gastric mucosal levels of IL-6 are elevated in *H. pylori*-associated gastritis and they decline after the infection is being treated. Meta-analyses further indicate that individuals infected with CagA-positive strains of *H. pylori* exhibit higher grades of inflammation than those infected with CagA^−^ strains [35]. Many studies have reported that the expression of IL-6 is affected by CagA; however, the underlying mechanism is unknown.

In the present study, MOI was optimized to investigate the efficiency of CagA in AGS cells with *H. pylori* coinfection. After *H. pylori* infection (MOI = 100), the CagA in the cytoplasm significantly enhanced the level of IL-6 mRNA and protein in AGS cells (Fig. 2a), which is consistent with previous reports [34–36]. We also observed that *CagA* knockout led to a decrease in the expression of IL-6, and that when combined with the silencing of eEF1A1, the expression of IL-6 further decreased. These results suggest that CagA-eEF1A1 co-mediates IL-6 expression (Fig. 2c). It should be recognized that since protein function and structure are interconnected, it may be beneficial to carry out additional functional studies on the CagA-eEF1A1 interaction to further elucidate the role of this complex in IL-6 regulation.

Considering that *H. pylori* has many virulence factors, we sought to exclude the influence of other factors on the expression of IL-6 by constructing and utilizing Ad-*cagA* to transduce AGS-C and AGS-sh*eEF1A1* cells. Our results, therefore, confirm that CagA plays a vial role in regulating IL-6 expression (Fig. 2d). Thus far, our results indicate that, following transduction, Ad-*cagA* promotes IL-6 expression through eEF1A1 in AGS cells only after treated with OSM. P-STAT3^S727^ can bind to NF-κB p65, thereby affecting the production of IL-6 and the development of inflammatory carcinoma [36]. Meanwhile, NF-κB has been found to be activated by *H. pylori*’s cagPAI [37]. We have experimentally shown that CagA increased NF-κB-p65 activity in the nucleus (Additional file 1: Figure S1). In this study, we also observed that CagA increased the level of p-STAT3^S727^ in the nucleus via interacting with eEF1A1, which may result in enhanced binding with NF-κB-p65 and subsequent promotion of *H. pylori* infection [38]. Moreover, we found that when eEF1A1 was reduced in cells, the activity of p-STAT3^S727^ in the nucleus did not increase, further suggesting that CagA-eEF1A1 co-mediates the expression of IL-6 by affecting the activity of p-STAT3^S727^7 in the nucleus.

PKCδ significantly impacts the phosphorylation level of STAT^S727^, while has no influence on the nuclear transport of STATS and tyrosine phosphorylation of residue 705. PKCδ can modulate p-STAT3^S727^ nucleation and affect its DNA binding and transcriptional activity by phosphorylating STAT^S727^ in the cytoplasm of cells [39]. Furthermore, PKCδ reportedly phosphorylates eEF1A1 threonine 431 [40, 41], and a strong regulatory effect has been demonstrated for eEF1A1-IL-6 in the gp130 receptor-mediated signalling pathway. Therefore, p-STAT3^S727^ were measured in this study to assess the activation of PKCδ. Herein, we found that cytoplasmic CagA can enhance eEF1A1 recruitment of PKCδ, which may reduce the inhibitory effect of PKCδ on p-STAT3^S727^, thereby increasing the concentration of nuclear p-STAT3^S727^, and strengthening the intranuclear cooperation between p-STAT3^S727^ and NF-κB-p65 to enhance the induction of IL-6 expression. We further confirmed that CagA enhances the recruitment of PKCδ via eEF1A1 (Fig. 4a), which subsequently increased the p-STAT3^S727^ content in the nucleus (Fig. 3) and enhanced the expression of IL-6. The PKCδ-blocking experiment further confirmed this inference (Fig. 4). Collectively, these data suggest that eEF1A1 plays an essential role in bridging CagA and PKCδ to induce IL-6 secretion and p-STAT3^S727^ accumulation.

**Fig. 5:**
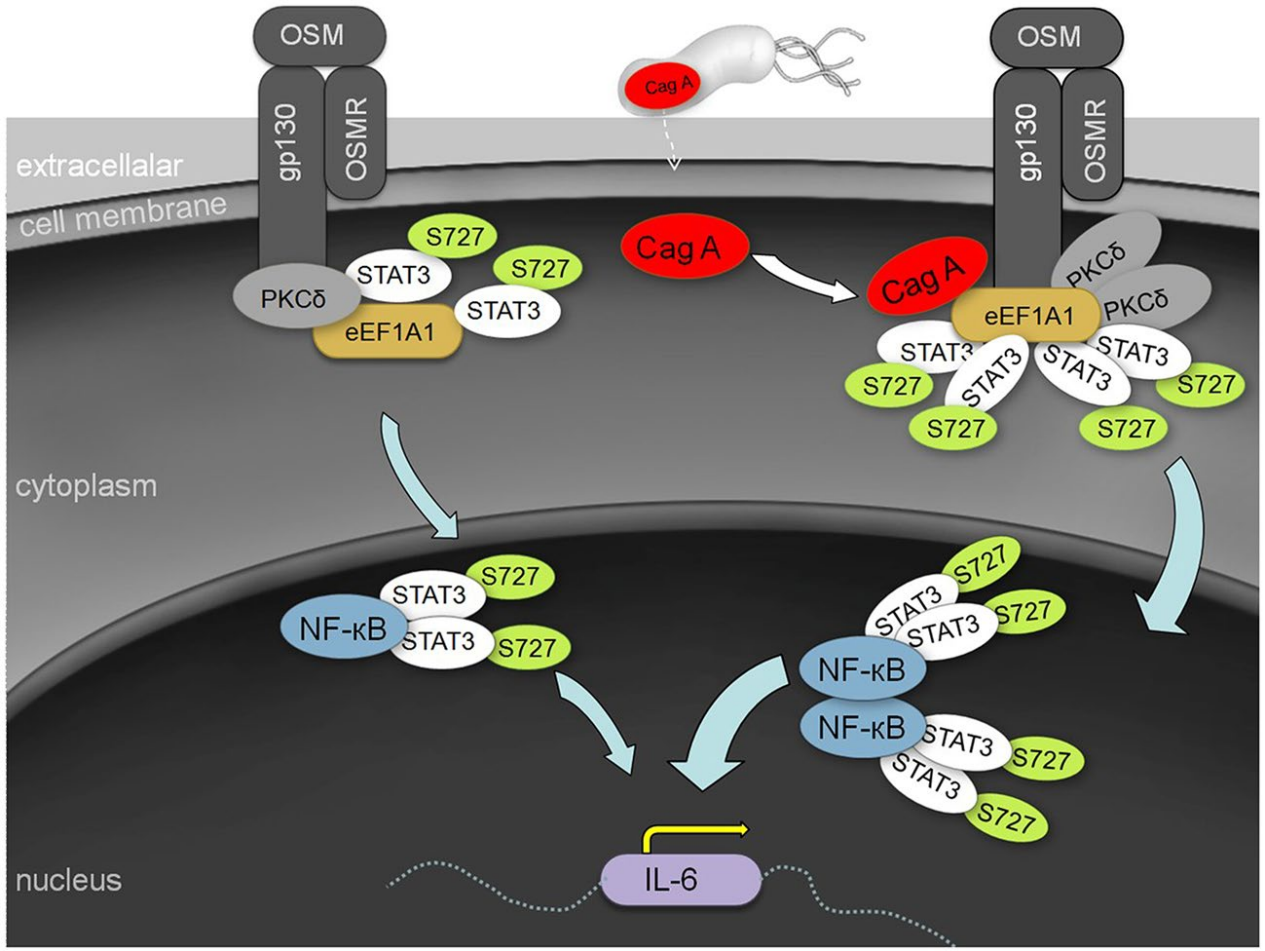
Postulated mechanism by which CagA promotes the expression of IL-6 in gastric cancer. CagA dysregulated the gp130/NF-κB/STAT3 pathway by recruiting PKCδ through eEF1A1 in the cytoplasm and increasing the accumulation of p-STAT3 ^S727^ in the nucleus. CagA, cytotoxin-associated gene A; eEF1A1, eukaryotic translation elongation factor 1-alpha 1; PKC, protein kinase C; IL-6, interleukin-6; STAT3, signal transducer and activator of transcription 3; gp130, glycoprotein 130; NF-κB, nuclear factor kappa-B

It has been clearly demonstrated that the JAK-STAT3 signalling pathway can affect tumour development by affecting the proliferation, survival, invasion, and immunosuppression of tumour cells, while also directly affecting tumour growth by affecting stromal cells to alter the inflammatory response or tumour microenvironment. Hence, STAT3 is expected to become a novel tumour therapeutic target [34]. In this study, CagA was found to clearly upregulate the expression of IL-6 by enhancing the recruitment of PKCδ via eEF1A1 in the cytoplasm, thereby increasing p-STAT3^S727^ phosphorylation in the nucleus. These results suggest that CagA-eEF1A1-PKCδ-p-STAT3^S727^-IL-6 serves as an important inflammatory pathway in *H. pylori*-related gastric cancer cells at the molecular level, unveiling the possible pathogenic mechanism of *H. pylori* infection in gastric cancer (Fig. 5). This mechanism implicates p-STAT3^S727^ as a potential novel therapeutic target for blocking *H. pylori* infection.

Therefore, this study may provide scientific basis for the prevention and treatment of gastric cancer, particularly that related to *H. pylori*. We also note that rottlerin may not be the most appropriate inhibitor of PKCδ [42] and further studies are needed to comprehensively validate this result. Moreover, further clarification is needed on the interactions between CagA and eEF1A1 to determine if additional proteins are required for the complex formation and to verify the abnormal regulation of other signalling pathways, inflammatory factors, and release of cytokines.

## Conclusions

In summary, CagA-M (residues 256‒871aa) was found to interact with eEF1A1-I (residues 1‒240aa) during *H. pylori* infection. CagA-eEF1A1 cooperated to mediate the expression of IL-6 by affecting the activity of p-STAT3^S727^ in the nucleus. Furthermore, CagA promoted IL-6 expression in AGS cells by recruiting PKCδ through eEF1A1 in the cytoplasm to increase the phosphorylation of STAT3^S727^in the nuclear. Moreover, the results indicated that CagA-mediated IL-6 production by forming CagA-eEF1A1-PKCδ complexes may promote *H. pylori* infection-induced gastric carcinogenesis. These findings provided new insights into the function of CagA-eEF1A1 in gastric tumorigenesis.

## Methods

### Materials

Anti-rabbit IgG HRP-linked antibody, anti-mouse IgG HRP-linked antibody, PKCδ (D10E2) rabbit mAb, phospho-Stat3 (Ser727) antibody, total STAT3 antibody, and Myc-Tag (9B11) mouse mAb were obtained from Cell Signalling Technology (Beverly, MA, USA). DMSO, monoclonal NTI-FLAG^®^M2 (clone M2) antibody, and anti-β-actin antibody were purchased from Sigma-Aldrich Co. (St. Louis, MO, USA). Protein A&G Plus-Agarose, normal rabbit IgG, normal mouse IgG, rabbit anti-CagA (b-300) antibody, mouse anti-CagA (A-10) antibody, and goat anti-rabbit IgG-AP were obtained from Santa Cruz Biotechnology (Santa Cruz, CA, USA). Protease/Phosphatase Inhibitor Cocktail (100 ×), anti-lamin B1 antibody, rottlerin anti-eEF1A1 antibody, and anti-GAPDH antibody were purchased from Abcam Inc (Cambridge, MA, USA). Protease inhibitor cocktail tablets were purchased from Roche Applied Science (Roche Diagnostics GmbH, Mannheim, Germany). Bicinchoninic acid (BCA) protein assay reagent was obtained from Pierce Biotechnology (Rockford, IL, USA). DMEM, DMEM/F12, FBS, and NE-PER Nuclear and Cytoplasmic Extraction Reagents were obtained from Thermo Scientific (Rockford, IL, USA). PE Annexin V Apoptosis Detection Kit I (BD Pharmingen^TM^) was obtained from BD Biosciences (Pharmingen, San Diego, CA, USA). Human OSM was obtained from Sinobiological Inc. Columbia blood agar base was provided by Oxoid Ltd (Basingstoke, Hampshire, England). The cell counting Kit-8 (CCK-8) was obtained from DOJINDO (Shanghai, China). All other chemicals used were in the purest form available commercially.

### Cell culture

AGS cells (ATCC number: CRL 1739, a human gastric adenocarcinoma epithelial cell line) were obtained from SGST.CN and cultured in DMEM/F12 containing 10% FBS. COS-7 (ATCC number: CRL-165) cells were obtained from SGST.CN and cultured in DMEM (Invitrogen, 12430) containing 10% FBS and 100 mM sodium pyruvate (Invitrogen, 113600700). Both cells were incubated at 37 °C in a humidified atmosphere with 5% CO_2_. AGS-sh*eEF1A1* and AGS-C cells were generated by transducing AGS cells with LV-*eEF1A1*-RNA (31585) or LV-CON077-RNA (CON077) (Shanghai Genechem Co., Ltd.) and selecting them by culturing with 0.5 µg/ml puromycin. The eEF1A1 target sequence was 5’-TCTCCAGGATGTCTACAAA-3′. AGS-sh*STAT3* and AGS-NC cells were generated by infecting AGS cells with Lv-STAT3-shRNA or Lv-con-shRNA (a gift from Dr. Min Zheng, Fujian Key Laboratory for Translational Research in Cancer and Neurodegenerative Diseases, Institute for Translational Medicine, Fujian Medical University) and selecting them with 0.5 µg/ml puromycin. The STAT3 target sequence was 5′-GCAAAGAATCACATGCCACTT-3′.

### Bacterial strains and infection conditions

CagA^+^*H. pylori* strain NCTC11637 and CagA^−^*H. pylori* strain NCTC11637ΔcagA (cagA-deleted mutant) were grown on Columbia blood agar base with 10% sheep blood at 37 °C using the Campy Container System (Thermo Scientific). Cells were infected with *H. pylori* up to multiplicities of infection of 100:1 for varying time points [43].

### Preparation of cytosolic and nuclear extracts from *H. pylori*-infected AGS cells

After *H. pylori* infection, AGS cells were harvested at the indicated time points. The cells were washed three times with ice-cold 1 × PBS, then scraped into 1 mL 1 × PBS, followed by centrifugation at 1700×*g* for 5 min at 4 °C. Pellets were resuspended in hypotonic buffer A [10 mM N-2-hydroxyethylpiperazine-N’-2-ethanesulfonic acid (pH 7.9), 1.5 mM MgCl_2_, 10 mM KCl, 0.5 mM DTT, and 0.2 mM PMSF] for 15 min on ice. Nonidet P-40 was then added to a final concentration of 0.1%, and the samples were incubated for less than 5 min. The mixture was then centrifuged at 6000×*g* for 5 min at 4 °C. The supernatant was collected as the cytosolic extract and stored at −80 °C. The pellets were washed twice with hypotonic buffer A and resuspended again in hypertonic buffer C [20 mM N-2-hydroxyethylpiperazine-N’-2-ethanesulfonic acid (pH 7.9), 20% glycerol, 420 mM NaCl, 1.5 mM MgCl_2_, 0.2 mM EDTA, 0.5 mM DTT, and 0.2 mM PMSF] for 1 h on ice and then centrifuged at 17,000×*g* for 15 min at 4 °C. The supernatant containing nuclear proteins was collected and stored at −80 °C. The protein concentrations of the cytosolic and nuclear extracts were determined using the BCA protein assay reagent.

### Western blot analysis

Equivalent amounts of proteins (10–30 µg) were subjected to electrophoresis on 8% or 12% SDS–polyacrylamide gels and transferred to PVDF membranes. The membranes were blocked in 5% fat-free dry milk in PBS containing 0.1% Tween 20 (PBST) for 1 h at room temperature. The membranes were then incubated with primary antibodies in 3% fat-free dry milk in PBS overnight at 4 °C. Membranes were washed in PBST, followed by incubation with 1:2000 or 1:5000 dilutions of the corresponding HRP-conjugated secondary antibodies for 1.5 h and again washed with PBST. Immunoreactions were visualised with an ECL chemiluminescent detection kit (Intron).

### Plasmid, Ad-GFP and Ad-*cagA* construction and virus transduced conditions

All enzymes used for cloning procedures were purchased from New England Biolabs (Ipswich, MA 01938-2732). The pcDNA3.1-Myc-*eEF1A1* and pcDNA3.1-Flag-*cagA* expression plasmids were constructed by standard molecular biology techniques. The optimized *cagA* gene fragment was inserted into an adenoviral vector (AdEasy XL System, Strategene) to construct the Ad-*cagA* adenovirus expressing CagA protein and control Ad-GFP virus. AGS-C and AGS-sh*eEF1A1* cells were transduced with Ad-*cagA* or Ad-GFP viruses (MOI = 30), respectively.

### Co-IP and western blot analysis

Briefly, cells were transfected with plasmids or infected as described above. Harvested cells were lysed on ice with lysis buffer. The lysates were incubated with primary antibodies and protein A/G Plus-Agarose beads overnight at 4 °C. The beads were washed three times with lysis buffer, and western blot analysis was performed, as described above. The following primary antibodies were used: anti-PKCδ (1:1000), anti-p-STAT3^S727^ (1:1000), anti-MYC (1:1000), anti-FLAG (1:1000), anti-lamin B1 (1:1000), anti-CagA (1:200), anti-eEF1A1 (1:5000), and anti-GAPDH (1:10000).

### Reverse transcriptase PCR (RT-PCR)

Total RNA was isolated from AGS cells using Trizol and reverse transcribed into complementary DNA using OligodT primers and random hexamer primers (Ex *Taq* Kit Takara) according to manufacturer’s instructions. RT-PCR was performed using the Real-Time PCR System (Applied Biosystems) following standard procedures. PCR conditions were 40 cycles at 95 °C for 30 s, 60 °C for 60 s, and 72 °C for 30 s. The primers used for RT-PCR are as follows: *eEF1A1* forward, 5′-TCTGGTTGGAATGGTGACAA -3′ and reverse, 5′-ACGAGTTGGTGGTA GGATGC -3′; *IL*-*6* forward, 5′-AGTGAGGAACAAGCCAGAGC -3′ and reverse, 5′-GAGGTG CCCATGCTACATTT -3′; *β*-*actin* forward, 5′-AGCCTCGCCTTTGCCGA-3′, and reverse, 5′-CTGGTGCCTGGGGCG-3 -3′. Amplified products were resolved by 2‒3% agarose gel electrophoresis, stained with SYBR Premix Ex *Taq* Kit (Takara), and visualised with the imagequant TM LAS 4000.

### Elisa

Supernatants were harvested and centrifuged at 1700×*g* for 5 min. Concentrations of IL-6 in cell culture supernatants were determined using the Valukine ELISA human IL-6 kit (R&D Systems, Minneapolis, MN, USA) according to the manufacturer’s instructions.

### Establishment of *eEF1A1*- and *STAT3*-knockdown AGS cells

AGS cells were transfected with the LV-*eEF1A1*-RNA shRNA plasmid (31585; target sequence: TCTCCAGGATGTCTACAAA) or CON077 (Hu6-MCS-Ubiquitin-EGFP-IRES-puromycin) for 48 h, and then puromycin was added to the cells at a concentration of 500 ng/mL to screen the transfected cells. The stably transfected STAT3-knockdown cells were transfected with the LV-STAT3-RNA shRNA plasmid (target sequence: 5′-GCAAAGAATCACATGCCACTT-3′) and then cultured with puromycin (500 ng/mL).

### Statistical analysis

Data are represented as the mean ± SD, and Student’s *t* test or ANOVA was used for statistical analyses with the GraphPad Prism 7.0 software (GraphPad Software, San Diego, CA, USA). Differences between groups were considered significant when the P-value was < 0.05.

## Supplementary information

**Additional file 1: Supplementary Fig. S1.** CagA promotes increased NF-κB-p65 activity in the nucleus. AGS-C and AGS-sh*eEF1A1* cells were infected with NCTC11637 or NCTC11637Δ*cagA* bacterial solutions (MOI = 100). Precipitates were analysed by WB to detect NF-κB-p50 and NF-κB-p65. *CagA* cytotoxin-associated gene A, *NF-κB-p50* nuclear factor kappa B-p50, *NF-κB-p65* nuclear factor kappa B-p65, *MOI* multiplicity of infection, *CON* control, *WB* western blot.

## Data Availability

All data generated or analysed during this study are included in this published article and its supplementary information files.
